# Indirect fitness benefits through extra‐pair mating are large for an inbred minority, but cannot explain widespread infidelity among red‐winged fairy‐wrens

**DOI:** 10.1111/evo.13684

**Published:** 2019-02-07

**Authors:** Wendy Lichtenauer, Martijn van de Pol, Andrew Cockburn, Lyanne Brouwer

**Affiliations:** ^1^ Division of Ecology and Evolution, Research School of Biology The Australian National University Canberra ACT 0200 Australia; ^2^ Department of Animal Ecology Netherlands Institute of Ecology (NIOO‐KNAW) Wageningen The Netherlands; ^3^ Department of Biology Utrecht University Utrecht The Netherlands; ^4^ Department of Animal Ecology and Physiology, Institute for Water and Wetland Research Radboud University Nijmegen The Netherlands

**Keywords:** Compatible genes, cooperative breeding, fitness, good genes, inbreeding avoidance, Malurus, pairwise relatedness

## Abstract

Extra‐pair paternity (EPP) has been suggested to improve the genetic quality of offspring, but evidence has been equivocal. Benefits of EPP may be only available to specific individuals or under certain conditions. Red‐winged fairy‐wrens have extremely high levels of EPP, suggesting fitness benefits might be large and available to most individuals. Furthermore, extreme philopatry commonly leads to incestuous social pairings, so inbreeding avoidance may be an important selection pressure. Here, we quantified the fitness benefits of EPP under varying conditions and across life‐stages. Extra‐pair offspring (EPO) did not appear to have higher fitness than within‐pair offspring (WPO), neither in poor years nor in the absence of helpers‐at‐the‐nest. However, EPP was beneficial for closely related social pairs, because inbred WPO suffered an overall 75% reduction in fitness. Inbreeding depression was nonlinear and reduced nestling body condition, first year survival and reproductive success. Our comprehensive study indicates that EPP should be favored for the 17% of females paired incestuously, but cannot explain the widespread infidelity in this species. Furthermore, our finding that fitness benefits of EPP only become apparent for a small part of the population could potentially explain the apparent absence of fitness differences in population wide comparisons of EPO and WPO.

Understanding genetic mating behaviors is crucial as it alters estimates of fitness and thus influences the evolution of such behaviors. Many bird species live in socially monogamous pairs, yet genetic monogamy is rare (Griffith et al. 2002). The widespread occurrence of extra‐pair paternity (EPP), whereby offspring are sired by a male other than the social partner, implies that EPP might be beneficial. For males, extra‐pair mating is expected to increase their reproductive success, but for females the benefits are less obvious. Females may benefit directly as extra‐pair males might provide them with additional food (Tryjanowski and Hromada 2005), parental care (Townsend et al. 2010), and/or protection against predators (Gray 1997; Eliassen and Jørgensen 2014). However, females may also gain indirect benefits through enhanced offspring quality. For example, it has been suggested that females enhance offspring fitness by seeking extra‐pair mates with more diverse (Brown 1997), better (‘good genes’, Hamilton and Zuk 1982), or more compatible genes (Zeh and Zeh 1996) than her social mate. In addition to seeking genetic improvements, extra‐pair mating might be used to avoid the cost of inbreeding when a female is paired to a closely related male (Blouin and Blouin 1988; Brooker et al. 1990; Pusey and Wolf 1996).

Despite the many mechanisms for potential indirect benefits of EPP, studies comparing fitness traits between extra‐pair offspring (EPO) and within‐pair offspring (WPO) in birds have found contrasting results. Some studies have found that EPO are fitter than WPO, manifested as higher growth rates, recruitment, or lifetime reproduction (e.g., Forstmeier et al. 2002; Foerster et al. 2003; Gerlach et al. 2012). However, other studies found that EPO have similar or even worse performance than WPO (Sardell et al. 2012; Moreno et al. 2013; Hsu et al. 2015). One explanation for the lack of evidence for indirect benefits could be that the advantage of being extra‐pair might only become apparent in specific situations. If only certain types of individuals in the population may benefit, for example females paired to inferior males (Brouwer et al. 2010), it may be hard to detect benefits of EPP in species where EPP is rare. Benefits might also only become apparent under unfavorable conditions (Schmoll et al. 2005; Arct et al. 2013) or during specific life‐stages (Gerlach et al. 2012; Hsu et al. 2017). Thus, to fully understand the potential indirect benefits of EPP for females, ideally fitness of EPO and WPO need to be compared in situations where benefits are expected in the first place.

Under the inbreeding avoidance hypothesis, EPO should not suffer the inbreeding depression that results from the expression of recessive deleterious alleles or perhaps from loci where heterozygous combinations are advantageous (Charlesworth and Charlesworth 1987; Hedrick and Garcia‐Dorado 2016). However, the benefits for EPP as an inbreeding avoidance mechanism might only become apparent when the fitness costs of inbreeding are strong or when inbreeding is frequent (Hajduk et al. 2018). Inbreeding is for example expected to be frequent in viscous populations, where many social pairs are formed by close relatives due to high philopatry of both sexes. Furthermore, inbreeding depression can affect different fitness traits (e.g., growth and reproductive success) throughout the lifespan of the individual (Keller and Waller 2002). However, most studies have focussed on early life, since inbreeding depression is likely to be less pronounced in adults because of selective disappearance of the most inbred individuals during the juvenile stage. On the other hand, weaker selection against deleterious alleles that act late in life might cause inbreeding depression to be more prevalent among the remaining inbred adults (Szulkin et al. 2013; Huisman et al. 2016). Thus, to determine whether extra‐pair mating serves as an inbreeding avoidance mechanism, one ideally determines the importance of inbreeding depression in fitness components at different life stages.

In addition to the assumptions that EPO should have a reduced degree of inbreeding and that there is inbreeding depression in the population, the inbreeding avoidance hypothesis also predicts that more closely related pairs should have higher EPP rates (Leclaire et al. 2013; Arct et al. 2015). To date these assumptions have only been tested comprehensively in song sparrows (*Melospiza melodia*; Taylor et al. 2010; Reid et al. 2015) and superb fairy‐wrens (*Malurus cyaneus*; Hajduk et al. 2018). However, we argue that for a complete understanding of EPP as an inbreeding avoidance mechanism, we also need to *quantify* the fitness benefits of EPP, as such quantifications are essential when determining the strength of selection (although indirectly as it affects the offspring) and thus the evolution of extra‐pair mating behavior.

Here, we present a comprehensive study that tests whether females gain indirect benefits from EPP in the cooperatively breeding red‐winged fairy‐wren (*Malurus elegans*). This is an excellent system to study this topic, because (i) the majority of female red‐winged fairy‐wrens gains EPP (Brouwer et al. 2011), without any indication of gaining direct benefits, suggesting there must be indirect benefits of EPP for the majority of the population. (ii) The limited dispersal of both sexes means that the probability of social pairing between relatives and thus the risk of inbreeding is high (Russell and Rowley 2000). (iii) At the same time, the limited dispersal means that key fitness components such as survival and recruitment are not confounded with undetected dispersal. (iv) Finally, large natural variation in the number of helpers per group and yearly breeding conditions mean that we can evaluate benefits of EPP under good and poor rearing conditions.

Previous work in red‐winged fairy‐wrens suggests that EPP plays a role in inbreeding avoidance, because more closely related social pairs have higher EPP and females gain paternity from extra‐pair mates that are less closely related to them than their social partner (Brouwer et al., 2011, 2014a). At the same time, the high levels of EPP mean that social context is no reliable cue for relatedness. Thus, by choosing an extra‐pair mate, females might inadvertently mate with a relative in such viscous populations. In addition, it remains unknown whether there actually is inbreeding depression or whether extra‐pair mating helps with avoiding it.

Using the red‐winged fairy‐wren as a model system, we aim to test the following predictions: First, under the ‘good genes’ hypothesis EPO are generally expected to outperform WPO, whereas if this is only true for siblings from mixed paternity broods (i.e., for females that are apparently paired to an incompatible male), this provides evidence for the compatible genes hypothesis. Second, if the benefits of extra‐pair paternity only become apparent under poor rearing conditions, we predict that EPO will outperform WPO when hatched in poor years (with lower than average reproductive success) or in the absence of helpers at the nest, because helped offspring are heavier and grow better, and larger offspring survive better in their first year of life (Brouwer et al. 2014b). Third, we predict that the high philopatry of both sexes results in frequent inbreeding and inbreeding depression. Fourth, if females paired to a highly related male use EPP to avoid the negative consequences of inbreeding, we expect that EPO will be less inbred, and predict that WPO of highly related social pairs perform worse than their EPO and also worse than offspring from unrelated pairs.

To compare performance of EPO and WPO, we use an integrated fitness measure that combines several fitness components: first year and adult survival, recruitment to a breeding position and reproductive success within the first three years of life. Such an integrated fitness measure accounts for the facts that some components of fitness are more important than others and that singling out specific life‐stages may under‐ or overestimate the overall benefits of EPP on lifetime fitness. In addition, to determine which underlying processes of inbreeding depression and benefits of EPP play a role, we examine nestling body condition and growth and each fitness component separately.

## Methods

### STUDY AREA AND DATA COLLECTION

Data were collected for seven cohorts of offspring hatched during the 2008 to 2014 seasons that were monitored up to and including 2016 in Smithbrook Nature Reserve, Western Australia (34°20′S, 116°10′E). The main study area comprised ∼65 territories in which > 99% of the adult birds were individually color‐banded. In this area, each territory was checked at least fortnightly for group composition, survival, and breeding activity throughout the breeding season (October–January). In addition, in another ∼30 territories surrounding the main area 80% of the birds were color‐banded and nest searching was done opportunistically. Social status was determined from behavioral observations, plumage variation, and age (Russell and Rowley 2000; Brouwer et al. 2011), with each group comprising a “dominant” pair‐bonded male and female and from zero to eight subordinate male and/or female helpers (Lejeune et al. 2016). Once located, nests were checked (at least) twice a week to collect data on number of eggs, hatchlings, and fledglings. Blood samples were taken when nestlings were ∼2 two days old and any unhatched eggs were collected for genotyping. Nestlings were colour‐banded, weighed and measured when they were ∼8 days old. Sex of birds was based on plumage characteristics for adults and DNA sexing of nestlings using P2/P8 primers (Griffiths et al. 1998).

Eighty‐eight percent of the border of the reserve is bounded by unsuitable habitat (farmland), but three narrow corridors lead away from the reserve allowing for dispersal to the surrounding state forests (Brouwer et al. 2014a). From 2009 onwards, each year 50–220 territories in the areas surrounding the study area were monitored opportunistically and checked for dispersers (up to 2‐km radius). The spatial configuration of our main study area and the fact that long‐distance dispersal is extremely rare (median dispersal distance = 150 m, L. Brouwer unpublished data), mean that we can accurately estimate survival consequences for both males and females (detection rate is virtually 100%) (Lejeune et al. 2016).

### PATERNITY AND INBREEDING

All blood (ca. 15 μL) and tissue samples were stored in 1 mL of 100% ethanol and stored at room temperature. Parentage was determined with high accuracy using 7 or 8 hypervariable microsatellite markers (mean: 30 alleles) with a parent‐pair analysis in program Cervus 3.0 (Kalinowski et al. 2007) as described in Brouwer et al. (2011). Samples from 1599 offspring from 717 broods were genotyped. Parentage (extra‐pair or within‐pair) could be determined for 98% of all sampled offspring (*N* = 1565 from 711 broods) and showed that 58% of offspring was extra‐pair (i.e., sired by another male than the dominant pair‐bonded male) and 67% of broods contained at least one EPO. For 93% of these offspring both parents were assigned with high accuracy (Brouwer et al. 2011), with most unassigned offspring due to incomplete sampling of adults in outer areas. In the main study area, both parents could be assigned for 99% of all offspring. Starvation of nestlings was negligible, but nest predation was high (∼70%). There was no evidence that unhatched eggs were more likely to be inbred or sired extra‐pair (Table B9).

Red‐winged fairy‐wren offspring of both sexes usually stay with their parents for at least one year to help raise the next brood. This means that despite that we have genotyped seven cohorts, sample sizes of known grandparents that are required to build up a pedigree are small. We used the microsatellite marker data to calculate pairwise relatedness (*r*), which measures the proportion of alleles shared by two individuals that are identical by descent (Keller and Waller 2002). Pairwise *r* of the genetic parents was used as an estimate of the inbreeding coefficient *f* ( = *r*/2) of the offspring, whereas pairwise *r* of the social parents was used to estimate the coefficient of kinship *k* ( = *r*/2) between social parents (Szulkin et al. 2013). Pairwise *r* was calculated according to Wang's method (2002) in the program KINGROUP v2 (Konovalov et al. 2004), as previous work has shown that this measure performs best for our highly polymorphic microsatellites (Brouwer et al. 2011). Note that these *r* values (and thus our *f* estimates) can become negative (Wang 2002).

Validating raw *r* values against the available pedigree of individuals with known relatedness showed that this measure performed well with mean *r* = 0.47 ± 0.002 S.E. for known first order relatives and *r* = 0.24 ± 0.001 S.E. for known second‐order relatives (see also Fig. A2). Methods exists to estimate an inbreeding coefficient *f* directly from the microsatellite data (Ritland 1996; Lynch and Ritland 1999) instead of using *f* = *r*/2, but this measure did not perform as well as pairwise *r*. Estimating *f* directly showed that two out of the five known inbred offspring's genetic parents had *r* = 0.36, close to the expect 0.375, whereas the offspring's *f* was only estimated as *f* = 0.02 (Lynch and Ritland 1999). For graphical purposes and to enable comparison of groups of individuals we binned *f* and *k* values as follows, based on *f* = 0.24 ± 0.03 S.D. for known mother‐offspring dyads: *f* (or *k*) ≈ 0.25 (*f* > 0.22), *f* ≈ 0.1875 (0.16< *f* ≤ 0.22), *f* ≈ 0.125 (0.10< *f* ≤ 0.16), *f* ≈ 0.0625 (0.04< *f* ≤ 0.10), *f* ≈ 0 (–0.02 < *f* ≤ 0.04), *f* ≤ –0.0625 (*f* ≤ –0.02). We considered offspring with *f*≳0.125 to be highly inbred.

### INDIVIDUAL FITNESS

For each offspring we calculated its fitness over the first three years of life. We chose to focus on the first three years, because (i) most birds that obtain a breeding position do so within that period (72%, 103 out of 143 individuals) and being recruited as a breeder is major determinant of fitness (Brouwer et al. in review), (ii) this allowed us to include four cohorts, while any extra year of life over which we would calculate fitness would reduce our sample size by one entire cohort.

In its simplest form individual fitness can be thought of as the number of gene copies an individual contributes to the next generation. If we initially focus on one time step, the contribution *W* of individual *i* to the next year is given by one's own survival (*J* = 0 or 1) plus half the number of offspring (*Y* = 0,1,2,…) an individual produces:

(1) $$W_{i}=J_{i}\;+\frac{1}{2}Y_{i}$$

However, this approach counts all surviving individuals equally, while in species with a clear stage‐structure—such as cooperatively breeding fairy wrens—some individuals have a much higher contribution to the future generations than others. To address this complexity Fisher (1930) developed the concept of reproductive value, which quantifies the contribution of individuals of a given state to the long‐term population growth rate.

Specifically, in the case of red‐winged fairy‐wrens the reproductive value of a surviving offspring is much lower than the reproductive value of a surviving parent (Brouwer et al. in review). This is because a surviving female offspring typically becomes a helper and does not reproduce independently in its second year of life, while a surviving dominant rarely loses its dominance status. Similarly, a surviving male offspring also typically becomes a helper and rarely gains any within‐group or extra‐pair paternity in their first year of life, though dominant males do routinely gain success in their next year. To account for the differences in reproductive value we weighted the contribution of the focal individual by the reproductive value of an individual that belongs to that sex and state (i.e., state typically being a dominant), and that of the offspring by the average reproductive value (*v*) of a surviving offspring (i.e., state typically being a helper) (Engen et al. 2009):

(2) $$W_{i}=J_{i}v_{\mathrm{state}\left(i\right),\mathrm{sex}\left(i\right)}\;+\frac{1}{2}\sum_{y=1}^{y=Y_{i}\;}J_{y}v_{\mathrm{state}\left(y\right),\mathrm{sex}\left(y\right)}$$

Now that we have derived above individual fitness measure over one year we can expand it to encompass multiple—in our case three—timesteps *t*:

(3) $$\begin{array}{ccc} W_{i,\;t_{0\;to\;3}} & = & J_{i,\;t_{0\;to\;3}}v_{\mathrm{state}\left(i,t_{3}\right),\mathrm{sex}\left(i\right)}\\ & & +\frac{1}{2}\sum_{t=0}^{t=3}\sum_{y=1}^{y=Y_{i,t}\;}J_{y,t}v_{\mathrm{state}\left(y,t\right),\mathrm{sex}\left(y\right)} \end{array}$$

In equation (3), $J_{i,\;t_{0\;to\;3}}$ denotes a binary variable of whether one survived the first three years of life or not, while $v_{state\left(i,t_{3}\right),sex\left(i\right)}$ denotes the state‐dependent reproductive value of the surviving individual after three years. Furthermore, the second term includes whether an offspring survived till the next year or not ($J_{y,t})$ multiplied by the reproductive value of that surviving offspring $\left(v_{state(y,t),sex(y)}\right)$, which is summed across all offspring produced by individual *i* in each of the three years.

(Brouwer et al. (in review)) already quantified the reproductive value of individuals that differed in state (helper or breeder) and sex (male or female) for this population, here we reiterate the rationale. We constructed a two‐sex demographic life‐cycle graph model that describes the main life‐history stages (dominants, subordinates, and fledglings). In this model the transitions between states are described by the following sex‐dependent fitness components: number of genetic fledglings produced, the fledging survival till the next breeding season; the annual adult survival for subordinates and dominants; the transition rates (conditional on survival) among states, that is the probability that a fledgling, subordinate or dominant will be a dominant the next year. The next step was to translate this life‐cycle graph into a matrix population model, in which the matrix elements consist of the fitness components that were derived from the field data as input to the projection matrix. Finally, we used standard matrix algebra to derive the state‐ and sex‐dependent reproductive values from the transition matrix (Caswell 2001). Reproductive values were scaled such that individual fitness is in units of reproductive value of a female dominant breeder (*v_D♀_*).

## Statistical Analyses

### INDIVIDUAL FITNESS

The distribution of individual fitness contains many zeros (most offspring die before ever producing any offspring of their own), and for individuals that do survive or produce offspring in the first three years of their life, individual fitness is a noninteger positive value due to the weighting with reproductive value. To model the bimodally distributed individual fitness variable we used a two‐part hurdle model. The first part modeled whether an individual had any fitness or not by fitting individual fitness as a binary variable (fitness = 0/fitness > 0) in a logistic linear‐mixed model. In the second part, for individuals with nonzero fitness their fitness was fitted in a linear mixed model using a Gaussian distribution. Both models contained mother and nest identity as nested random effects, to account for non‐independence of the data. The joint likelihood of both parts of the hurdle model was used for model inference using a model selection approach (see below).

We tested whether EPO outperformed WPO, or whether this is only true under poor rearing conditions, by including the following fixed factors/covariates as main effects and their interactions with EPP (whether an offspring was sired extra‐pair (yes/no)) in both parts of the hurdle model: quality of hatch year (yearly mean number of fledglings per group), number of helpers at the nest, and *k* of the social parents. In addition, models were also built to investigate the possibility that variation in fitness is better described by a nonlinear (i.e., threshold) relationship, by creating three binary‐fixed factors for whether or not offspring were: extra‐pair and hatched in a poor quality year; extra‐pair and reared without helpers at the nest; within‐pair from closely related social pairs (related at the level of a half‐sib or higher). There were two poor cohort years (2009 and 2011; fledgling production 0.97 and 1.13, respectively) and two good years (2008 and 2010; fledgling production 1.58 and 1.69, respectively). Although female helpers benefit offspring growth more than male helpers (Brouwer et al. 2014b), there was no indication that helpers of both sexes affected fitness differently (Fig. A1) and thus we only modeled the total number of helpers to minimize the number of parameters in the model.

Subsequently, to investigate the role of inbreeding on individual fitness a similar hurdle model was used to asses the relationship between fitness and inbreeding, by including either the inbreeding coefficient as a linear covariate, or by including whether the offspring was inbred (*f* ≳ 0.125) or not as fixed categorical factor (effectively modeling a nonlinear threshold function of *f*).

### FITNESS COMPONENTS

To investigate potential underlying processes leading to fitness benefits of EPP and/or inbreeding depression, we examined two measures of offspring quality and four fitness components separately. Using General(ized) Linear Mixed Models ((G)LMMs), first year survival after fledging, adult survival (from one to three years of age) and recruitment to a breeding position (within first three years of life) were fitted as a binary variable (yes/no) using a logit link function, whereas nestling body condition (mass accounting for size) and growth (tarsus size) were fitted using a Gaussian distribution and identity link. Reproductive success (number of genetic fledglings produced within first three years of life) was fitted using a zero‐inflated Poisson distribution. Nestling fitness components were analyzed with nest identity and mother identity as (nested) random intercepts. For first year survival and the adult fitness components only mother identity was included as a random intercept as most females only fledge a single nest per season and thus no additional variation could be explained by nest identity. For analyses on recruitment probability and reproduction only those individuals that survived until three years of age were included, to avoid bias due to mortality before having the chance to recruit/reproduce. Data availability varied between the traits of interest (see for details Table A1).

The role of EPP (yes/no), inbreeding coefficient and whether an offspring was inbred (*f* ≳ 0.125) or not, was investigated by including them as fixed factors/covariates. In addition, where necessary we controlled for the following fixed variables (see Tables Appendix B): brood size (range 1–3), experience of the breeding female (first time breeder, yes/no), nestling age (in days, log‐transformed), day of season the nestling was measured (DoS, starting from October 1), nestling tarsus size, the number of male helpers, number of female helpers, sex, and quality of the year of hatching (annual mean number of fledglings produced per group). In addition, nestling body condition (residuals from a linear regression of tarsus and age on body mass) and relative nestling body size (residuals from a linear regression of age on tarsus size) were accounted for in analyses of first year survival to be able to distinguish whether potential inbreeding depression acted directly on survival or via condition or growth of the young. To test whether inbred offspring benefit more from being extra‐pair, all juvenile fitness components included an interaction between EPP and inbreeding coefficient or between EPP and whether or not an offspring was inbred (*f* ≳0.125). These interactions could not be tested for the adult fitness components, due to the high mortality, and thus low sample sizes, of inbred individuals.

To analyze individual fitness and the fitness components, we used a model selection approach to find the most parsimonious models, using Akaike's information criterion corrected for sample size (Akaike 1973; Burnham and Anderson 2002). Models that are better supported by the data result in lower AIC_c_ values. We used an all‐subset approach with all possible combinations of predictors (Appendix B), except that predictors in their linear form were not included simultaneously with their nonlinear (threshold) form. All covariates were scaled to z‐scores before including in the models.

In addition to using the full datasets for each fitness component, we investigated whether there was evidence for the compatible genes hypothesis by performing paired comparisons of EPO and WPO from the same nest (maternal half‐sibs). This was done for individual fitness, nestling condition and size and first year survival, and has the additional advantage that it reduces the potential bias due to confounding variables (because the offspring experience the same environment). For these paired analyses non‐parametric tests were performed. All statistical analyses were performed in R3.4.0 (R Development Core Team 2017) using RStudio (RStudio Team 2016). Binomial and Gaussian distributions were fitted using package lme4 (Bates et al. 2015), zero‐inflated Poisson models were fitted using glmmTMB (Brooks et al. 2017). Model selection was carried out using package MuMIn (Bartoń 2016).

## Results

### ARE THERE FITNESS BENEFITS OF EPP?

**Figure 1:**
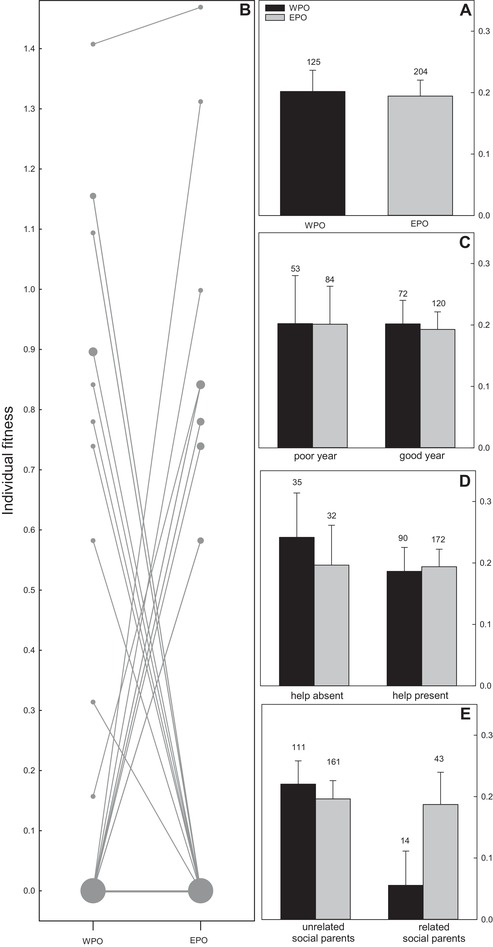
Individual fitness within the first three years of life of within‐pair offspring (WPO) and extra‐pair offspring (EPO) of red‐winged fairy‐wrens for (A) all individuals, (B) maternal half‐sib (*N* = 56 sibpairs from 42 nests, 35 sibpairs both had zero fitness), (C) offspring hatched during “poor” (below average reproduction) and “good” (above average reproduction) years, (D) in the absence and presence of helpers‐at‐the‐nest and (E) unrelated and related social parents (at the level of half‐sibs). Reproductive values were scaled such that individual fitness is in units of reproductive value of a female dominant breeder (*v_D♀_*). Note that in (B) lines connect WPO and EPO from the same brood and the thickness of the symbols indicates the sample sizes with many maternal‐half sibs comparisons both having zero fitness. Error bars represent SE.

Our results show that there is no evidence for the “good” genes hypothesis, because EPO did not appear to be fitter than WPO in the first three years of life (Fig. 1A): including EPP as a predictor of variation in individual fitness was not supported by the data (∆AICc = +2.2; Table B1 model 20 vs 1). Our results also failed to support the compatible genes hypothesis, as EPO did not appear to have higher fitness than their maternal half‐sibs from the same brood (Fig. 1B; Wilcoxon V = 82.8, *P* = 0.78, *N* = 42 half‐sib pairs). In addition, there was no evidence that EPO outperformed WPO under poor rearing conditions: EPO did not appear to have higher fitness than WPO when hatched in a poor year (Fig. 1C; ∆AICc = +1.0, Table B1, model 3 vs 1) or in the absence of helpers at the nest (Fig. 1d; ∆AICc = +1.1, Table B1 model 17 vs 1). However, in accordance with the inbreeding avoidance hypothesis, WPO of close kin (*k* ≳0.125) did have much lower fitness than all other offspring (Fig. 1E), this association was consistently included in the top models and accounted for 52% of the Akaike model weight (∆AICc = –1.0; Table B1, model 1 vs 4).

### IS THE RISK OF INBREEDING HIGH?

The risk of inbreeding was high, as due to the limited dispersal of both sexes, 17% of social pairings were between close kin (*k* ≳ 0.125; 53 of 310 pairs), and 8% between first‐order relatives (*k* ≈ 0.25; 24 of 310). However, inbreeding itself occurred less frequently, as only 7% of all assigned offspring were the result of inbreeding at the level of half‐sibs or higher (*f* ≳ 0.125; 106 of 1458 offspring; 68 of 680 (10%) broods) and inbreeding between first‐order relatives was even more rare: 1% of all assigned offspring (*f* ≈ 0.25; 14 of 1458; 8 of 680 (1.2%) broods). A full breakdown of rates of inbreeding and inbred individuals can be found in Table A3.

### DOES EPP REDUCE INBREEDING?

**Figure 2:**
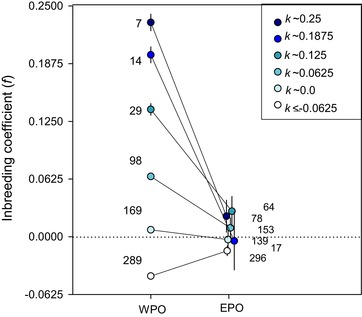
Inbreeding coefficient (*f*) of within‐pair offspring (WPO) and extra‐pair offspring (EPO) given for varying values of kinship of the social parents (*k*). Numbers indicate sample sizes, error bars represent 95% confidence intervals around means. Dotted line indicates *f* = 0.

In accordance with the inbreeding avoidance hypothesis, EPO were less inbred than WPO when considering offspring of social pairs that were related to the degree of cousins or higher (*k* ≳ 0.0625, Fig. 2). On average the inbreeding coefficient of the EPO was close to zero (mean ± SE: 0.015 ± 0.004), whereas logically the inbreeding coefficient of WPO mirrored *k* (Fig. 2). Despite the finding that EPP reduced inbreeding, 50% (7 out of 14) of the first‐order inbred offspring were the result of extra‐pair mating, and all of these were sired by males from other social groups.

### IS THERE INBREEDING DEPRESSION?

**Figure 3:**
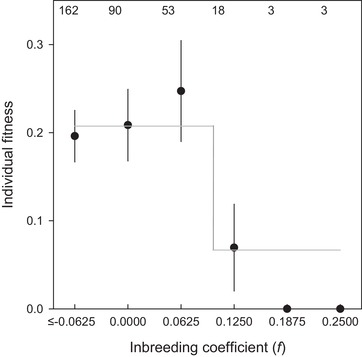
The relationship between the inbreeding coefficient (*f*) and individual fitness of individuals in their first three years of life. Individual fitness is in units of reproductive value of a female dominant breeder (_D_♀). Numbers on top indicate sample sizes. Error bars represent ± SE. Line shows fit of the top model (Table B2).

Our results show strong inbreeding depression: inbred individuals (*f* ≳ 0.125) had ∼ 75% lower fitness than less inbred individuals (Fig. 3; individual fitness: 0.05 + 0.04 vs. 0.21 + 0.02, respectively). This association received considerable support as all top models included one of the two predictors of inbreeding (model without inbreeding: ∆AICc = +2.5, Table B2, model 4 vs model 1). The fitness reduction depended nonlinearly on inbreeding, as only offspring inbred at the level of half‐sibs or more suffered from fitness reductions (Fig. 3); indeed whether or not an individual was inbred was a better predictor than a linear effect of the inbreeding coefficient (∆AICc = –3.0, Table B2, model 1 vs model 5).

### WHAT ARE THE UNDERLYING PROCESSES?

The apparent absence of an overall difference in fitness between EPO and WPO (Fig. 1A) was not due to different fitness components being affected in opposing directions, as including EPP as a predictor for variation in fledgling survival, adult survival, recruitment, and reproductive success were not supported by the data (Tables B3–6). Similarly, maternal half‐sibs did not differ in any of the separate fitness components (Table A2).

**Figure 4:**
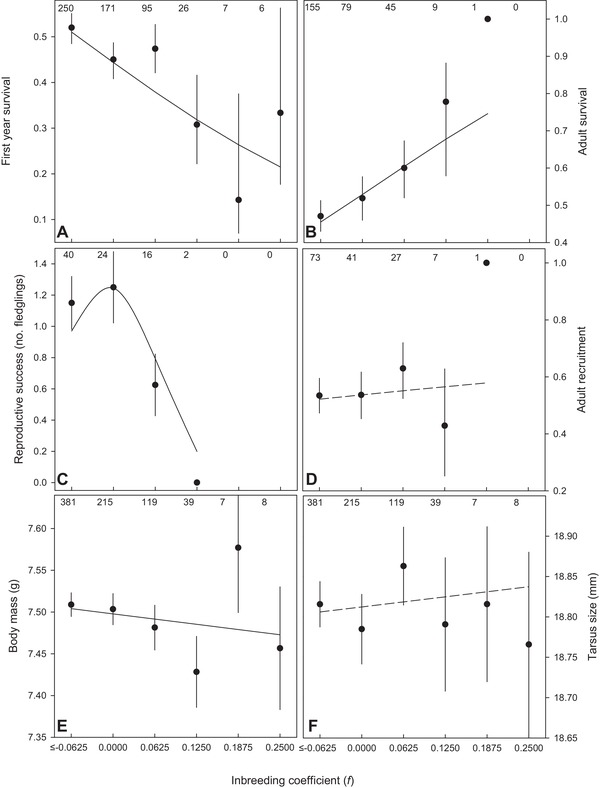
The relationship between inbreeding coefficient (*f*) and (A) first year survival from fledging, (B) adult survival from one to three years of age, (C) reproductive success (number of genetic fledglings), (D) recruitment to a breeding position within the first three years of life, (E) nestling body mass and (F) nestling size (tarsus length). Numbers on top indicate sample sizes. Error bars represent ± SE. Lines show trendlines derived from the effects sizes of *f* on each fitness component (Tables B3–8), with solid lines indicating an association was supported by the data.

The negative association between the inbreeding coefficient and individual fitness was mediated through inbreeding depression at different stages in life. Early in life inbred fledglings had lower chances of surviving their first year (Fig. 4A), and this association was strongly supported by the data (∆AICc = –5.0, Table B3, model 366 vs. 1). Later in life there were mixed effects from being inbred: there was considerable evidence that more inbred adults survived better between one and three years of age (Fig. 4B; Table B4, ∆AICc = –1.7, model 5 vs 1, models with inbred or inbreeding coefficient accounted for 81% of the Akaike model weight). At the same time there was some evidence that these individuals were less likely to reproduce successfully than noninbred individuals (Fig. 4C), although this model was only moderately supported, possibly due to low sample sizes of highly inbred adults (∆AICc = –0.7, Table B5, model 3 vs 1). Lower reproductive success was not the result from less opportunity to reproduce, as there was no evidence that surviving inbred individuals had a lower chance of obtaining a dominant position (Fig. 4D, Table B6, ∆AICc = +1.8, model 4 vs 1).

Inbreeding depression acted directly on body condition as inbred nestlings had lower condition (mass after accounting for size; Fig. 4E; ∆AICc = –3.5, Table B7, model 10 vs 1). In contrast, there was no evidence that inbreeding reduced structural growth (tarsus size) of nestlings (Fig. 4F, ∆AICc = +2.0, Table B8, model 1 vs 8). There was no evidence that inbreeding depression in first year survival acted through effects of body condition or size, as the inbreeding coefficient was a better predictor for variation in survival than condition or size (∆AICc >+0.7, models 21 & 6 vs 1, Table B3).

## Discussion

Extra‐pair mating in birds is extremely widespread, but often there is no evidence females obtain direct benefits from the extra‐group males with which they mate. This suggests that females might gain indirect benefits from extra‐pair paternity (EPP) by enhancing the genetic make‐up of their offspring. However, many studies have failed to show that extra‐pair offspring (EPO) outperform within‐pair offspring (WPO) in population wide comparisons, perhaps because benefits might only become apparent during certain life‐stages, under specific conditions or because inbreeding is rare or its negative effects weak, making it hard to detect potential benefits. In red‐winged fairy‐wrens (*Malurus elegans*), a species in which the majority of females gain EPP and 17% of social pairs were closely related, overall EPO did not outperform WPO (Fig. 1A). However, for females socially paired to a closely related male the potential benefits from EPP were large, as their EPO were much less inbred (Fig. 2). Inbred offspring had 75% lower fitness, providing strong (indirect) selection against inbreeding (Fig. 3). Inbreeding depression occurred during both the juvenile and the adult phase (Fig. 4). Inbred offspring had lower chances of surviving their first year of life and were less likely to reproduce, which was not negated by the surprising finding that inbred adults survived better. Despite the finding that EPP can result in large benefits for females paired to a closely related male, no benefits were detected for the majority of the population, which nonetheless routinely engage in extra‐pair mating.

### INDIRECT FITNESS BENEFITS THROUGH EPP

Our results support the inbreeding avoidance hypothesis: females paired to close kin were able to decrease inbreeding depression through EPP. In addition, previous work showed that *M. elegans* females paired to a highly related male are more likely to have EPP and gain this from males that are less closely related to them than their social partner (Brouwer et al. 2011). Given our complete sampling and the fact that unhatched eggs were not likely to be more inbred or sired by EP males (Table B9), we are confident that this was not stemming from inbreeding depression in early offspring survival (Reid et al. 2015). The idea that EPP helps reduce inbreeding for highly related social pairs is also supported by a recent comparative study on nine fairy‐, emu‐, and grasswren species, which showed that incestuous social pairs always have higher EPP rates in the Maluridae genus (Brouwer et al. 2017).

Notwithstanding, to date for only two species the assumptions of the inbreeding avoidance hypothesis– the presence of inbreeding depression in the population; a reduction of inbreeding through EPP; and higher EPP rates for closely related pairs—have been comprehensively confirmed (Taylor et al. 2010; Reid et al. 2015; Hajduk et al. 2018). Our study now adds a third species to the literature. Moreover, our quantitative fitness estimation also shows that EPO from closely related pairs have 75% higher fitness than their WPO, thus EPP in closely related pairs should be favored and selection for EPP is very strong. In addition, our study shows that the fitness benefits only become apparent for offspring inbred at the level of half‐sib or higher. This means that only 17% of females benefited from EPP, whereas ∼67% of females gained EPP. These findings suggest that the costs of engaging in extra‐pair mating behavior are minimal for fairy‐wrens. A possible explanation is that the presence of helpers compensates for the risk associated with a reduction of investment in offspring care when males suspect they have been cuckolded (Mulder et al. 1994; Brouwer et al. 2017). Although it should be noted that the high frequency of EPP in the population itself potentially reduces relatedness among potential mates and thus the population as a whole might already benefit from excessive extra‐pair mating (Cornell and Tregenza 2007; Power and Holman 2014; Germain et al. 2018).

Despite the fact that extra‐pair mating can help reduce inbreeding, half of the highly inbred offspring (between first‐order relatives) were the result of extra‐pair mating. This is perhaps not surprising, since the high EPP rates mean that many females are unaware of the identity of their genetic father. In two of these cases, inbreeding was indeed the result of females dispersing to a breeding vacancy in their sire's territory. Together with the finding that the majority of females do not gain indirect benefits, this suggests that inbreeding avoidance is thus unlikely to be the main explanation for the occurrence of EPP in our study species.

It has previously been argued that EPP allows for the formation of incestuous social pairs (Cockburn et al. 2013) and under this scenario, there is little cost to incestuous pairings as both sexes can continue to produce noninbred offspring through extra‐pair mating. Alternatively, EPP has also been suggested to be a by‐product of selection on male mating behavior (Forstmeier et al. 2014). Contradicting this idea is that female red‐winged fairy‐wrens are consistent in their choice of their EP sire and prefer males that are able to moult into their nuptial plumage early in the season (Brouwer et al. 2011), most likely a reliable cue for male quality as early moulting is costly (Peters et al. 2000). Furthermore, radio‐telemetry in the superb fairy‐wren revealed that females fly directly to the male's singing post on his territory, indicating that females initiate extra‐pair fertilizations (Double and Cockburn 2000).

Since there were no detectable benefits for EPO when either considering all offspring or when comparing maternal haf‐sibs form the same brood, our study did not provide evidence for the “good” or compatible genes hypotheses (Hamilton and Zuk 1982; Zeh and Zeh 1996). Furthermore, EPO did appear to have higher fitness when reared under poor conditions. However, in our study we considered fitness components during the first three years of life, and although most benefits of EPP have been shown to occur during the juvenile stage (Suter et al. 2007; Wells et al. 2017), it is possible that certain benefits of EPP only become apparent later on. For example, timing of moulting into nuptial plumage is thought to be an honest signal of male quality for female extra‐pair mate choice (Dunn and Cockburn 1999; Brouwer et al. 2011), and is most variable among older males (van de Pol et al. 2012). If early acquisition of nuptial plumage is heritable, there could potentially be reproductive benefits particularly later in life when variance between males increases.

### INBREEDING AND THE UNDERLYING PROCESSES OF INBREEDING DEPRESSION

We found that 7% of all offspring were inbred at the level of half‐sib mating or higher (and 17% of the social pairs were close kin at that same level). Although such inbreeding level might seem low considering the fact that dispersal is low, longer distance dispersal does occur in *M. elegans* (i.e., five territories, Russell and Rowley 2000) and such movements have been shown to have large effects on reducing incestuous pairing in other species (Nelson‐Flower et al. 2012). Combined with the high rates of EPP, which are even higher for closely related pairs (Brouwer et al 2011), dispersal is apparently sufficient to reduce the chances of incestuous pairing. Nevertheless, our inbreeding estimates are much higher compared to other species. For example, inbreeding to the level of half‐sib mating or higher accounted for only 1% of all pairings in collared flycatchers (*Ficedula albicollis*) and for 2% of all nestlings in savannah sparrows (*Passerculus sandwichensis*) (Kruuk et al. 2002; Wheelwright et al. 2006). Both of the aforementioned populations were isolated island populations, with restrictions to gene flow. Comparable levels of inbreeding are only reported for a small island population of song sparrows (7.6%; (Keller 1998), indicating that philopatry of both sexes in *M. elegans* increases the chance of inbreeding to the level found in small insular populations.

Not surprisingly, the level of inbreeding in *M. elegans* was also much higher than in the closely related *M. cyaneus* (∼0.3%, Hajduk et al. 2018), a species where only males are philopatric. Routine inbreeding is expected to reduce inbreeding depression, as recessive deleterious alleles are expected to be purged from the population (Charlesworth and Willis 2009). However, despite the higher frequency of inbreeding, *elegans* showed greater inbreeding depression than *cyaneus*, where only nestling mass, and not survival, was negatively associated with inbreeding (Hajduk et al. 2018). This suggests that either the 7% frequency of inbreeding in *elegans* is still too low to reduce the frequency of deleterious alleles or that inbreeding depression acts through cumulative effects of multiple mildly deleterious mutations that are not easily purged from the population (Wang et al. 1999). Alternatively, in their study Hajduk et al. (2018) examined inbreeding depression in survival of offspring that are still being fed by the adults in their group, whereas the costs of inbreeding might become apparent in the period when young have to forage independently.

Interestingly, inbreeding depression did not gradually increase with inbreeding, but followed a threshold pattern, with very strong fitness reductions for highly inbred individuals, whereas inbreeding at the level of lower than half‐sibs did not have fitness consequences. Such patterns can occur when multiple genes interact (reinforcing epistasis, Lynch and Walsh 1998) and although rarely reported in wild populations, have been found in studies on dairy cattle (Wall et al. 2005; Gulisija et al. 2007), horses (Klemetsdal 1998) and *Drosophila melanogaster* (Sharp and Agrawal 2016).

In addition to inbreeding depression early in life, inbred adults were less likely to successfully raise a brood to fledging, and this was not caused by inbred adults having less opportunity due to lower chances of obtaining a dominant position. Inbreeding depression has often been found to reduce fertility, for example through reduced male attractiveness (Pilakouta and Smiseth 2017; Vega‐Trejo et al. 2017) or reduced parental care behaviors, such as female incubation attentiveness (Pooley et al. 2014). Future work will have to show what the underlying mechanisms of the lower reproductive success of inbred individuals are.

Surprisingly, inbred adults survived better than noninbred adults. This could simply result from the inbred individuals not being exposed to the cost of raising fledglings (since they produced fewer offspring), although this is rather unlikely as even though helpers of both sexes and dominant males might not have sired offspring, they routinely take care of young on their home territory. A few other studies have reported positive associations between inbreeding and fitness components, which were suggested to be the result of these offspring inheriting a “proven genotype” (i.e., high quality genotype) from a highly inbred parent (Weiser et al. 2016) or by purging and partial dominance of deleterious alleles (Moreno et al. 2015). Despite the limited dispersal in our study population, purging is extremely unlikely as there is no evidence for a recent bottleneck. To test the “proven genotype” idea we will need more data on multiple generations to investigate whether parents of the successful inbred individuals were also inbred.

## Conclusion

Our study shows that in a highly philopatric species, the risk of inbreeding was high and resulted in strong inbreeding depression. Both early and late life fitness components were affected and unexpectedly, inbreeding was also positively associated with a fitness component, stressing the importance of considering an integrated fitness measure to determine the presence and strength of selection. Despite the fact that no benefits of EPP were detected for the majority of the population, females paired to a closely related male were able to substantially increase their offspring's fitness through EPP, indicating strong selection for EPP in those pairs. This can explain why incestuous social pairs in red‐winged fairy‐wrens have the highest EPP levels. However, while indirect selection should favor EPP for the 17% of the females paired to a close relative, the cost of inbreeding cannot be a complete explanation for infidelity, as most females gain EPP. Possibly, our finding that only females paired to a close relative benefited from EPP might be more widespread and can explain the apparent absence of indirect benefits of EPP in other studies, where closely related pairs may be rare.

Associate Editor: I. Lovette

Handling Editor: Mohamed A. F. Noor

## Supporting information


**Table A1**. Details of Datasets used for analyses on effect of EPP and inbreeding in red‐winged fairy‐wrens for genotyped cohorts 2008‐2014 monitored up to and including 2016.
**Table A2**. Summary statistics of pairwise comparisons between EP and WP maternal half‐sibs from the same brood. In nests with multiple EPO or WPO, one pair was chosen at random.
**Table A3**. Rates of inbreeding and number of inbred individuals. The number and percentage of social pairs, broods and offspring per binned inbreeding coefficient are given.
**Figure A1**. Individual fitness (± S.E.) within the first three years of life of within‐pair offspring (WPO) and extra‐pair offspring (EPO) of red‐winged fairy‐wrens in a.) the absence and presence of female helpers‐at‐the‐nest and in b.) the absence and presence of male helpers‐at‐the‐nest.
**Figure A2**. Mean (± S.E.) inbreeding coefficient (*f* = *r*/2) for known relationships between dyads of a) grandparent‐grandoffspring and half‐siblings (*f* ≈ 0.125) and for b) parent‐offspring and full‐siblings (*f* ≈ 0.25).

**Appendix B**: Model selection tables for individual fitness, first year survival, adult survival, adult reproductive success, adult recruitment, nestling body condition, nestling size and hatching probability.
