# Supplementary material for: Indirect fitness benefits through extra‐pair mating are large for an inbred minority, but cannot explain widespread infidelity among red‐winged fairy‐wrens
Source: Evolution. 2019 Feb 7;73(3):467–80. doi: 10.1111/evo.13684 (PMC7172280; doi:10.1111/evo.13684)
Supplement: Supplementary file 1 — Table A1. Details of Datasets used for analyses on effect of EPP and inbreeding in red‐winged fairy‐wrens for genotyped cohorts 2008‐2014 monitored up to and including 2016. Table A2. Summary statistics of pairwise comparisons between EP and WP maternal half‐sibs from the same brood. In nests with multiple EPO or WPO, one pair was chosen at random. Table A3. Rates of inbreeding and number of inbred individuals. The number and percentage of social pairs, broods and offspring per binned inbreeding coefficient are given. Figure A1. Individual fitness (± S.E.) within the first three years of life of within‐pair offspring (WPO) and extra‐pair offspring (EPO) of red‐winged fairy‐wrens in a.) the absence and presence of female helpers‐at‐the‐nest and in b.) the absence and presence of male helpers‐at‐the‐nest. Figure A2. Mean (± S.E.) inbreeding coefficient (f = r/2) for known relationships between dyads of a) grandparent‐grandoffspring and half‐siblings (f ≈ 0.125) and for b) parent‐offspring and full‐siblings (f ≈ 0.25). [file EVO-73-467-s001.docx]

# Appendix A.

**Table A1.** Datasets used for analyses on effect of EPP and inbreeding in red-winged fairy-wrens for genotyped cohorts 2008-2014 monitored up to and including 2016.

| Predictor | Cohorts | Sample size | Details |
| --- | --- | --- | --- |
| *Nestling body condition and size* | 2009-2014 | 769 | Mass and size of offspring in the nest. Breeding experience of dominant female unknown for 2008 cohort. |
| *First year survival* | 2008-2014 | 555 | First year survival of fledged offspring. |
| *Adult survival* | 2008-2013 | 289 | Survival for 2^nd^ and 3^rd^ year of life. |
| *Recruitment* | 2008-2013 | 149 | Recruitment to a breeding position within the first three years of life, conditional upon survival |
| *Reproductive success* | 2008-2011 | 82 | Genetic offspring produced by adults in 2^nd^ and 3^rd^ year of life. |
| *Individual fitness* | 2008-2011 | 329 | For first three years of life, since fledging. |

**Table A2**. Summary statistics of pairwise comparisons between EP and WP maternal half-sibs from the same brood. In nests with multiple EPO or WPO, one pair was chosen at random. To compensate for the possible influence of this random selection, the tests were repeated 10^4^ times and the average test statistic and p-value are reported.

| Trait | EPO vs. WPO ± SE | Test statistic | N | *P* |
| --- | --- | --- | --- | --- |
| *Individual fitness* | 0.19 ± 0.06 vs. 0.18 ± 0.05 | Wilcoxon signed rank V: 82.77 | 42 | 0.78 |
| *Nestling body condition* | 7.74 ± 0.10 vs. 7.53 ± 0.12 | Paired t: -1.39 | 92 | 0.18 |
| *Nestling tarsus size* | 19.42 ± 0.29 vs. 19.10 ± 0.30 | Paired t: -0.31 | 92 | 0.73 |
| *First year survival* | 0.51 (0.45 – 0.56) vs. 0.44 (0.39 – 0.50) | McNemar χ^2^: 0.83 | 64 | 0.42 |

**Table A3**. Rates of inbreeding and inbred individuals. The number and percentage of social pairs, broods and offspring per binned inbreeding index values are given.

| Binned *f* value | Social pairs | Broods | Offspring |
| --- | --- | --- | --- |
| *-0.0625* | 121 (39.0%) | 399 (58.7%) | 739 (50.7%) |
| *0* | 79 (25.5%) | 234 (34.4%) | 396 (27.2%) |
| *0.0625* | 57 (18.4%) | 133 (19.6%) | 217 (14.9%) |
| *0.125* | 20 (6.5%) | 49 (7.2%) | 71 (4.9%) |
| *0.1875* | 9 (2.9%) | 12 (1.8%) | 21 (1.4%) |
| *0.25* | 24 (7.7%) | 8 (1.2%) | 14 (1.0%) |
| Total | 310 | 680 | 1458 |


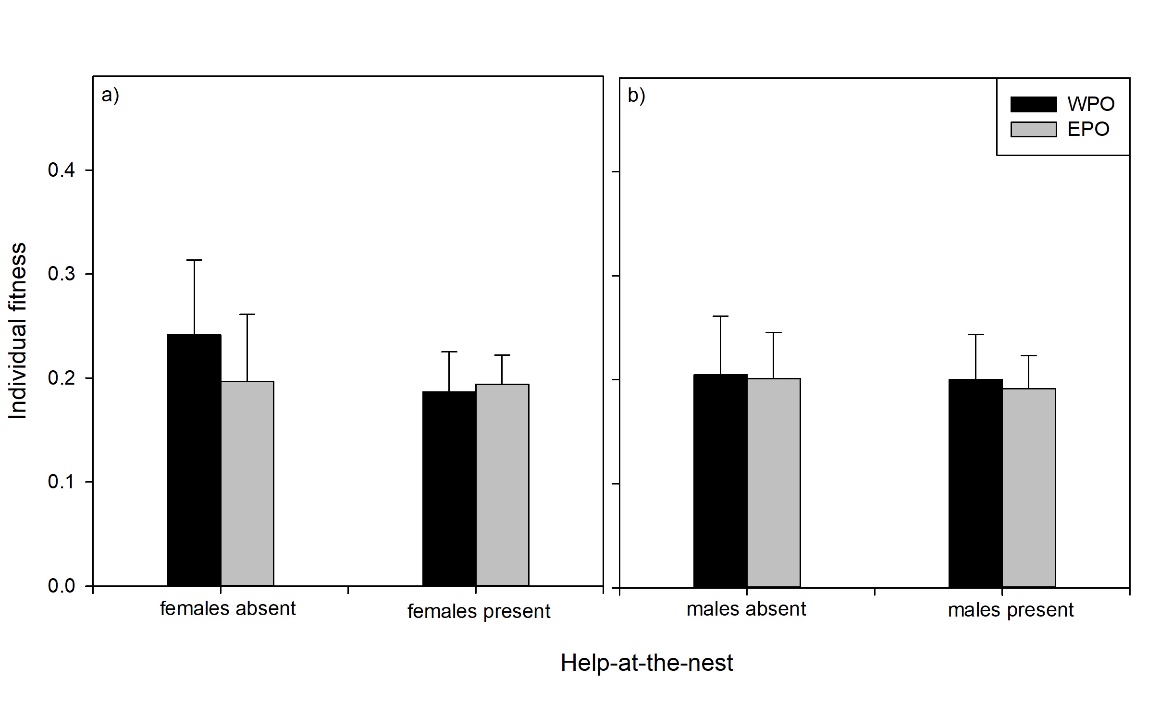


**Figure A1.** Individual fitness (± S.E.) within the first three years of life of within-pair offspring (WPO) and extra-pair offspring (EPO) of red-winged fairy-wrens in a.) the absence and presence of female helpers-at-the-nest and in b.) the absence and presence of male helpers-at-the-nest.


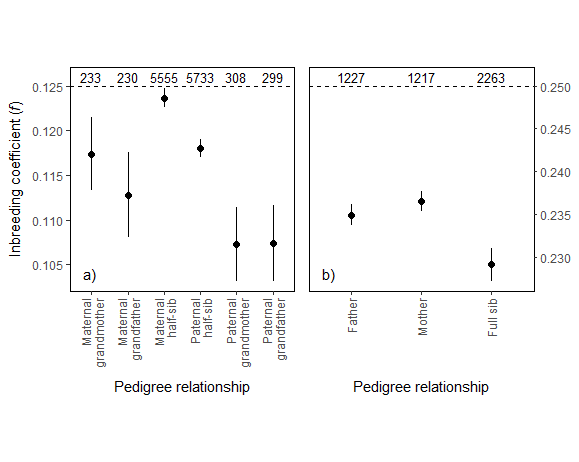

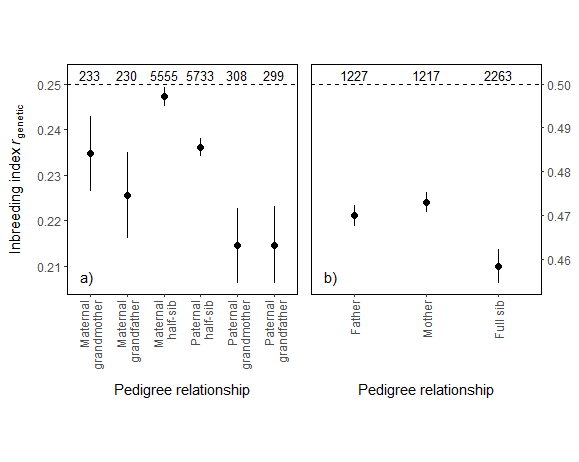


**Figure A2**. Mean (± S.E.) inbreeding coefficient (*f* = *r*/2) for known relationships between dyads of a) grandparent-grandoffspring and half-siblings (*f* ≈ 0.125) and for b) parent-offspring and full-siblings (*f* ≈ 0.25). Numbers on top represent sample sizes.
